# Supplementary material for: Identification of photoperiod-induced specific miRNAs in the adrenal glands of Sunite sheep (Ovis aries)
Source: Front Vet Sci. 2022 Jul 22;9:888207. doi: 10.3389/fvets.2022.888207 (PMC9354845; doi:10.3389/fvets.2022.888207)
Supplement: Supplementary file 1 [file Data_Sheet_1.ZIP › Supplementary file/miRDeep2 file.zip!/miRDeep2 file/Know_Structure/SPLP42A1/oar-mir-127.pdf]

miRBase precursor : oar-mir-127
Total read count : 117793
oar-miR-127 read count : 115874
remaining reads : 1919

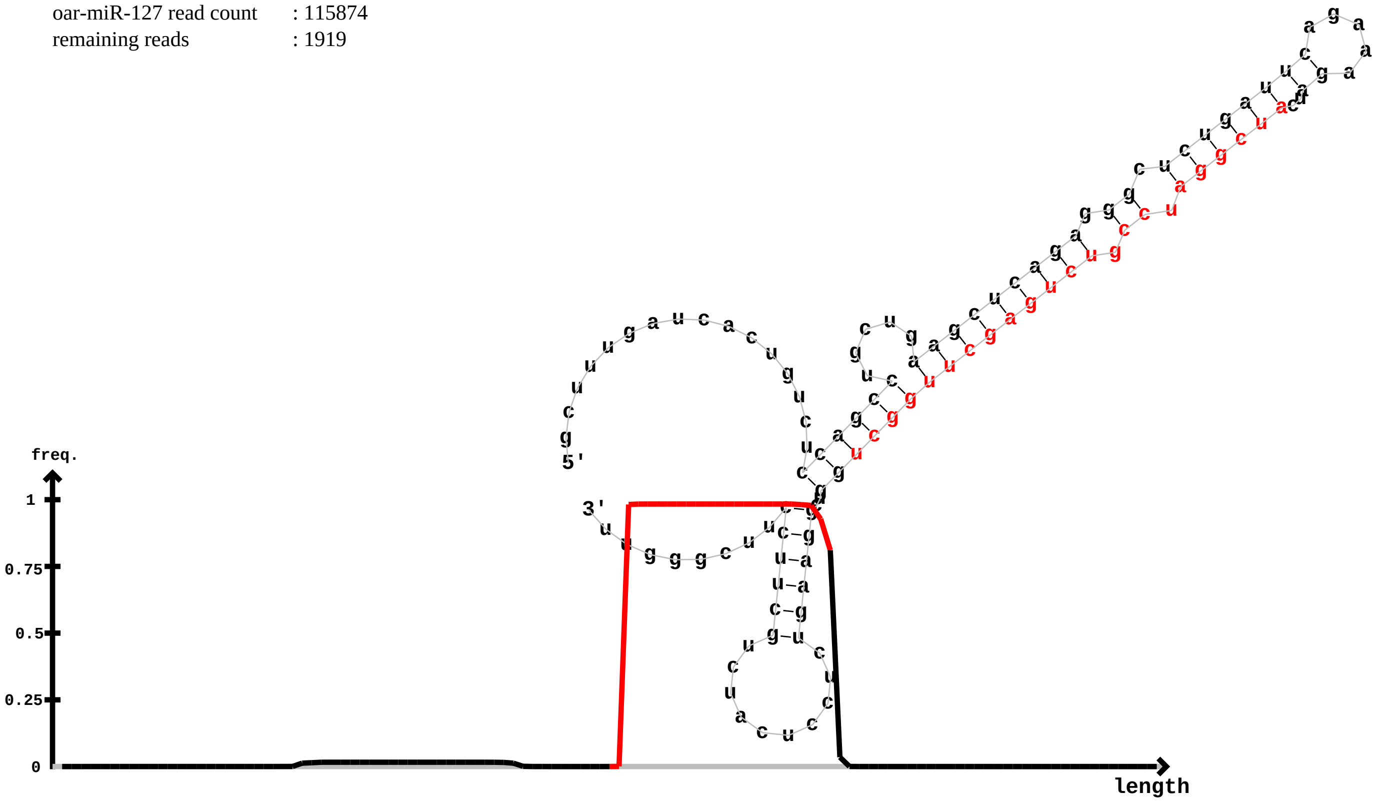

oar-miR-127

```
5'- gcuuugaucacugucuccagccugcugaagcucagagggcucugauucagaaagaucaucggauccgucugagcuuggcuggucggaagucuccucaucugcuuccucggguu  -3'    exp
    ...............(((((.....(((((((((.((.((((((((.....))..)))))).)).))))))))))))))..((((((.........))))))...........   reads   mm   sample
    gcuuugaucacugucuccagccug.........................................................................................   2       0    seq
    .cuuugaucacugucuccagccug.........................................................................................   2       0    seq
    ..uuugaucacugucuccagccug.........................................................................................   2       0    seq
    ....ugaucacugucuccagccug.........................................................................................   3       0    seq
    ......................ugcugaagcucagagggcucug.....................................................................   5       0    seq
    ......................ugcugaagcucagagggcucuga....................................................................   11      0    seq
    ......................ugcugaagcucagagggcucugau...................................................................   2       0    seq
    .......................gcugaagcucagagggcucu......................................................................   1       0    seq
    .......................gcugaagcucagagggcucuga....................................................................   2       0    seq
    .......................gcugaagcucagagggcucugau...................................................................   5       0    seq
    ........................cugaagcucagagggcA........................................................................   1       1    seq
    ........................cugaagcucagagggcuc.......................................................................   1       0    seq
    ........................cugaagcucagagggcucC......................................................................   1       1    seq
    ........................cugaagcucagagggcucug.....................................................................   9       0    seq
    ........................cugaagcucagagggcucuga....................................................................   59      0    seq
    ........................cugaagcucagagggcucugau...................................................................   312     0    seq
    ........................cugaagcucagagggcucugauu..................................................................   1070    0    seq
    ........................cugaagcucagagggcucugauuc.................................................................   21      0    seq
    ........................cugaagcucagagggcucugauucagaa.............................................................   1       0    seq
    .........................ugaagcucagagggcucug.....................................................................   1       0    seq
    .........................ugaagcucagagggcucuga....................................................................   4       0    seq
    .........................ugaagcucagagggcucugau...................................................................   9       0    seq
    .........................ugaagcucagagggcucugauu..................................................................   170     0    seq
    .........................ugaagcucagagggcucugauuc.................................................................   3       0    seq
    ..........................gaagcucagagggcucugau...................................................................   4       0    seq
    ..........................gaagcucagagggcucugauu..................................................................   136     0    seq
    ..........................gaagcucagagggcucugauuc.................................................................   69      0    seq
    ..........................gaagcucagagggcucugauuca................................................................   1       0    seq
    ..........................gaagcucagagggcucugauucag...............................................................   4       0    seq
    ...........................aagcucagagggcucugauu..................................................................   3       0    seq
    ...........................aagcucagagggcucugauucag...............................................................   3       0    seq
    ...........................aagcucagagggcucugauucaga..............................................................   1       0    seq
    ...............................................cagaaagaucaucggauccgucugagcuuggc..................................   1       0    seq
    .......................................................ucaucggauccgucugagcuuggc..................................   1       0    seq
    ........................................................caucggauccgucugagcuugg...................................   1       0    seq
```

oar-miR-127

```
gcuuugaucacugucuccagccugcugaagcucagagggcucugauucagaaagaucaucggauccgucugagcuuggcuggucggaagucuccucaucugcuuccuucggguu
........................................................caucggauccgucugagcuugg....................................   1       0       seq
........................................................caucggauccgucugagcuuggc...................................   1       0       seq
........................................................caucggauccgucugagcuuggcu..................................   2       0       seq
.........................................................aucggauccgucugagcuugg....................................   21      0       seq
.........................................................aucggauccgucugagcuuggc...................................   36      0       seq
.........................................................aucggauccgucugagcuuggcu..................................   1       0       seq
..........................................................ucggauccgucugagcu.......................................   7       0       seq
..........................................................ucggauccgucugagcuu......................................   262     0       seq
..........................................................ucggauccgucugagcuug.....................................   327     0       seq
..........................................................ucggauccgucugagcuugg....................................   5979    0       seq
..........................................................ucggauccgucugagcuuggc...................................   13712   0       seq
..........................................................ucggauccgucugagcuuggcu..................................   91227   0       seq
..........................................................ucggauccgucugagcuuggcug.................................   4055    0       seq
..........................................................ucggauccgucugagcuuggcugg................................   35      0       seq
..........................................................ucggauccgucugagcuuggcuggu...............................   2       0       seq
...........................................................cggauccgucugagcuu......................................   1       0       seq
...........................................................cggauccgucugagcuug.....................................   1       0       seq
...........................................................cggauccgucugagcuugg....................................   13      0       seq
...........................................................cggauccgucugagcuuggc...................................   16      0       seq
...........................................................cggauccgucugagcuuggcu..................................   129     0       seq
...........................................................cggauccgucugagcuuggcug.................................   31      0       seq
............................................................ggauccgucugagcuuggc...................................   3       0       seq
............................................................ggauccgucugagcuuggcu..................................   8       0       seq
............................................................ggauccgucugagcuuggcug.................................   2       0       seq
```
